# The Role of Vitamin D and Vitamin D Receptor in Immunity to *Leishmania major* Infection

**DOI:** 10.1155/2012/134645

**Published:** 2011-10-11

**Authors:** James P. Whitcomb, Mary DeAgostino, Mark Ballentine, Jun Fu, Martin Tenniswood, JoEllen Welsh, Margherita Cantorna, Mary Ann McDowell

**Affiliations:** ^1^Eck Institute for Global Health, Department of Biological Sciences, University of Notre Dame, Notre Dame, IN 46556, USA; ^2^Cancer Research Center, University at Albany, 1 Discovery Drive, Rensselaer, NY 12144, USA; ^3^Department of Veterinary and Biomedical Sciences, Pennsylvania State University, University Park, PA 16802, USA

## Abstract

Vitamin D signaling modulates a variety of immune responses. Here, we assessed the role of vitamin D in immunity to experimental leishmaniasis infection in vitamin D receptor-deficient mice (VDRKO). We observed that VDRKO mice on a genetically resistant background have decreased *Leishmania major*-induced lesion development compared to wild-type (WT) mice; additionally, parasite loads in infected dermis were significantly lower at the height of infection. Enzymatic depletion of the active form of vitamin D mimics the ablation of VDR resulting in an increased resistance to *L. major*. Conversely, VDRKO or vitamin D-deficient mice on the susceptible Th2-biased background had no change in susceptibility. These studies indicate vitamin D deficiency, either through the ablation of VDR or elimination of its ligand, 1,25D3, leads to an increase resistance to *L. major* infection but only in a host that is predisposed for Th-1 immune responses.

## 1. Introduction

The initial metabolism of vitamin D_3_ occurs in the skin where 7-dehydrocholesterol is converted to previtamin D_3_ after exposure to UVB radiation. After isomerization, vitamin D_3_ is metabolized by hepatic 25-hydroxylase (CYP2R1) to form 25-hydroxyvitamin D_3_ (25OHD3), which is the major circulating form of vitamin D_3_. 25OHD_3_ is metabolized by 1*α*-hydroxylase (CYP27B1) that is present in the kidney and many other target tissues including the skin. The active form of vitamin D_3_, 1*α*,25 dihydroxyvitamin D3 (1,25D3) translocates to the nucleus of target cells, where it binds to the vitamin D receptor (VDR), a member of the nuclear receptor supergene family. The VDR heterodimerizes with the retinoid X receptor and this complex recognizes vitamin D response elements in the promoters of many genes to modulate their transcription.

1,25D3 is well characterized for its function in maintaining appropriate serum calcium concentrations as well as its critical requirement for proper bone formation. In addition to these roles, 1,25D3 is important in the regulation of immune responses. Vitamin D has important roles in leukocyte differentiation, dendritic cell maturation, and modulation of the T-helper cell dichotomy [1–5]. Symptoms of Th-1-mediated autoimmune diseases can be reduced or eliminated by treatment with 1,25D3 [6–8]. In addition to its role in autoimmune disease, recent studies have explored a role for vitamin D-mediated signaling in infectious disease resistance [9]. For example, *Mycobacterium tuberculosis* infection induces both VDR and 1-*α* hydroxylase, which are necessary for production of cathelicidin as part of an antimicrobial peptide response against the *M. tuberculosis* infection in vitro [10]. Here, we investigated the role of VDR in immunity to murine experimental cutaneous leishmaniasis (CL), the prototypical *in vivo* model of the Th-1/Th-2 dichotomy. Parasites from the genus *Leishmania* are responsible for a spectrum of diseases ranging from self-healing cutaneous disease to the potentially fatal visceral disease. Immunity to *L. major, *a parasite responsible for cutaneous leishmaniasis, is highly dependent on a strong IFN*γ*-mediated T-helper 1 (Th-1) response. This Th-1 response induces production of nitric oxide (NO) which is critical in elimination of parasites. 

We report that VDRKO mice exhibit decreased lesion development as well as decreased parasite loads at the height of *L. major *infection. Additionally, we demonstrate that resistant mice that are rendered vitamin D-deficient by ablation of the 1-*α* hydroxylase enzyme in CYP27B1 KO mice mimic VDRKO mice with regards to decreased lesion development, suggesting that the effect is dependent on the presence of the ligand. Surprisingly, deficiency of vitamin D signaling in genetically susceptible mice has no effect on *L. major* infection.

## 2. Materials and Methods

### 2.1. Mice

 VDRKO [11], CYP27B1KO [12], and WT mice, on both BALB/c and C57BL/6 backgrounds, were maintained at Friemann Life Sciences Center at the University of Notre Dame (Notre Dame, Ind, USA). To generate the BALB/c VDRKO strain, female C57BL/6 VDRKO mice were bred with WT male BALB/c mice. The female offspring from this mating were genotyped by PCR [11] and the mice identified as heterozygous for the VDRKO allele were backcrossed to WT male BALB/c mice. This process was repeated 7 times resulting in mice with a greater than 99.2% BALB/c background. Male and female heterozygous mice were bred to each other to generate offspring that were homozygous for either the VDRKO allele or for the WT allele. These homozygous mice were then used to establish a breeding colony of both VDRKO and WT mice on a BALB/c background. VDRKO and WT mice were maintained from weaning on a high-calcium, high-lactose rescue diet (TD96348; Teklad, Madison, Wis, USA) to prevent hypocalcemia associated with VDR deficiency [13]. All animal protocols were approved and reviewed by the University of Notre Dame Institutional Animal Care and Use Committee (IACUC). All mice used in the experiments described below were females between 1 and 4 months of age. WT female mice were age-matched for all experiments.

### 2.2. Parasites and Infection

*L. major* NIH Friedlin V1 strain (MHOM/IL/80/FN) was used in this study. The parasites were cultivated and infective stage, metacyclic promastigotes were generated as previously described [14]. For infections, 1 × 10^5^ metacyclic promastigotes in 20 *μ*L of PBS were injected intradermally into the outside surface of the ear. Lesion diameter and thickness and ulcer diameter was measured weekly with a digital vernier caliper. A lesion “score” was calculated by adding the values obtained for lesion diameter, ulcer diameter, and lesion thickness. Infected ear tissue was homogenized to determine relative parasite load by performing a limiting dilution assay as previously described [15].

### 2.3. Histology

Segments of ear tissue were embedded in Tissue-Tek OCT freezing media (Fisher Scientific, Pittsburgh, PA), sectioned and stained with Mayer's hematoxylin and eosin to distinguish cytoplasm and nuclei, respectively. Images were obtained using a Nikon E400 light microscope (Nikon, Melville, NY, USA).

### 2.4. IgG Subclass Determination

Collected sera was used in a soluble *Leishmania* antigen (SLA) specific ELISA. IgG subclass specific secondary antibodies (Southern Biotech, Birmingham, Ala, USA) were used to determine IgG subclass levels. Absorbances were recorded using a SpectraMax M2 plate reader (Molecular Devices, Sunnyvale, Calif, USA). Sera from mice 12 weeks after post-infection were pooled and run as a normalizing sample on each plate. For each isotype, the absorbance for each normalizing sample was used to generate a mean absorbance (ABS). Plate to plate variation was eliminated by using the equation (mean ABS/plate ABS) × (plate ABS) = normalized ABS.

### 2.5. Quantitative RT-PCR Analysis

Total RNA was isolated from pulverized ears using an RNeasy Mini kit (Qiagen, Valencia, Calif, USA) according to the manufacturer's instructions and contaminating DNA was removed via DNase I treatment (Invitrogen, Carlsbad, Calif, USA). Reverse transcription was performed using 1 *μ*g of DNA-free total RNA, 250 ng random primers (Invitrogen), and SuperScript III kit (Invitrogen). Real-time PCR was performed on an ABI 7900HT sequence detection analyzer (Applied Biosystems, Foster City, Calif, USA) using the 2x SYBR Green Kit (Applied Biosystems). The primers (300 nM; IDT, Coralville, Iolua, USA) used were HPRT, IFN*γ*, IL-4, IL-12p40 [16], Arg1 [17], iNOS [18], IL-10: 5′-CAC AAA GCA GCC TTG CAG AA-3′/ 5′-CTG GCC CCT GCT GAT CCT-3′, TNF*α*: 5′-GAA ACA CAA GAT GCT GGG ACA GT-3′/ 5′-CAT TCG AGG CTC CAG TGA ATT C-3′. For FoxP3, gene expression was determined using a premade gene expression assay (Applied Biosystems) according to manufacturer's instructions. Relative copy number was determined using the comparative CT method [19].

### 2.6. Restimulation Assay

Lymph nodes from infected mice were disrupted using a syringe plunger and a cell strainer (BD Biosciences, San Jose, Calif, USA) and cells from 4 mice per condition were pooled and stimulated with 50 *μ*g of SLA. Cell supernatant was harvested at 120 hr after treatment and was analyzed on a Luminex 200 instrument using a multiplex biomarker immunoassay (Millipore, Billerica, Mass, USA). Two biological replicates were assessed, the first in quadruplicate on two separate Luminex plates and the second in duplicate on one plate.

### 2.7. Flow Cytometry

FcReceptors were blocked with 10% normal mouse serum and stained using 1% BSA in PBS. Cell surface labeling was performed using the following antimouse antibodies: *α*-CD45, *α*-CD11b, *α*-CD11c, *α*-LY-6C/G, *α*-CD4, *α*-CD8, *α*-TCR (BD Biosciences), *α*-F4/80 (Invitrogen), and *α*-FoxP3 (eBioscience, San Diego, Calif, USA). Appropriate isotype controls were used as negative controls. Flow cytometry was performed using an FC-500 flow cytometer (Beckman Coulter, Fullerton, CA) and analyzed with MXP analysis software (Beckman Coulter).

### 2.8. Diet Studies

For vitamin D deficiency experiments, mice were fed TD.04179, a vitamin D-deficient diet (Harlan Teklad) from birth prior to mating with male mice from the same background. Once weaned, the offspring from these mice were fed solely using the vitamin D-deficient diet throughout the study. Mice were infected in the ears and lesions were measured as previously described. Vitamin D analysis of serum was performed using both a 25(OH)D3 ELISA and a 1,25D3 ELISA (IDS, Fountain Hills, AZ, USA) following the manufacturer's instructions.

### 2.9. Generation of Bone Marrow Derived Macrophages and Bone Marrow-Derived Dendritic Cells (DC)

Bone marrow was flushed from femurs with RPMI-C. Red blood cells were lysed using ice-cold sterile ACK lysis buffer (0.15 M Nh_4_Cl, 10 mM KHCO_3_, 0.1 mM Na_2_EDTA). Macrophages were generated as previously described [14]. For the generation of DC, progenitor cells were counted and resuspended at 2 × 10^5^/mL in RPMI-C containing 20 ng/mL granulocyte macrophage colony-stimulating factor (GM-CSF) (Peprotech, Rocky Hill, NJ, USA). The cells were supplemented with fresh RPMI-C containing 20 ng/mL GM-CSF on days 3, 6, 8, and 10. On day 12, the cells were transferred to 24 well plates for the duration of the experiment.

### 2.10. Phagocytosis Assays

Macrophages or DC were treated with IFN-*γ* (500 U/mL) and/or 1,25D3 (Biomol, Plymouth Meeting, Pa, USA) (40 nM) for 24 hr or left untreated. Cells were infected at a ratio of 5 : 1 with V1 strain *L. major* metacyclic promastigotes. Parasites were opsonized with 5% normal mouse serum for 30 minutes, washed, then coincubated with the macrophages or dendritic cells for 1, 2, 4, and 24 hr. Coverslips were stained with Diff-Quick (Fisher Scientific).

### 2.11. IL-12 ELISA/Nitric Oxide (NO) Assays

Levels of IL-12 were analyzed using anti-IL-12p40 ELISA (Pierce, Rockford, Ill, USA). NO production was assessed by Griess reaction according to the manufacturer's instructions (Promega, Madison, Wis, USA).

### 2.12. Statistical Analyses

For statistical analysis of lesion size, analysis of variance (ANOVA) between WT and KO strains was utilized with a subsequent Bonferonni's post-test to determine at what time points the strains were different. Paired *t*-tests were performed to determine differences between WT and KO populations for parasite burdens, IgG levels, cytokine regulation, and all *in vitro *analyses. All statistical analyses were performed using GraphPad Prism 4.0 software. In all cases, a *P* value ≤0.05 was considered statistically significant. 

## 3. Results

### 3.1. VDRKO Mice on a C57BL/6 Background Exhibit Increased Resistance to L. major Infection

**Figure 1:**
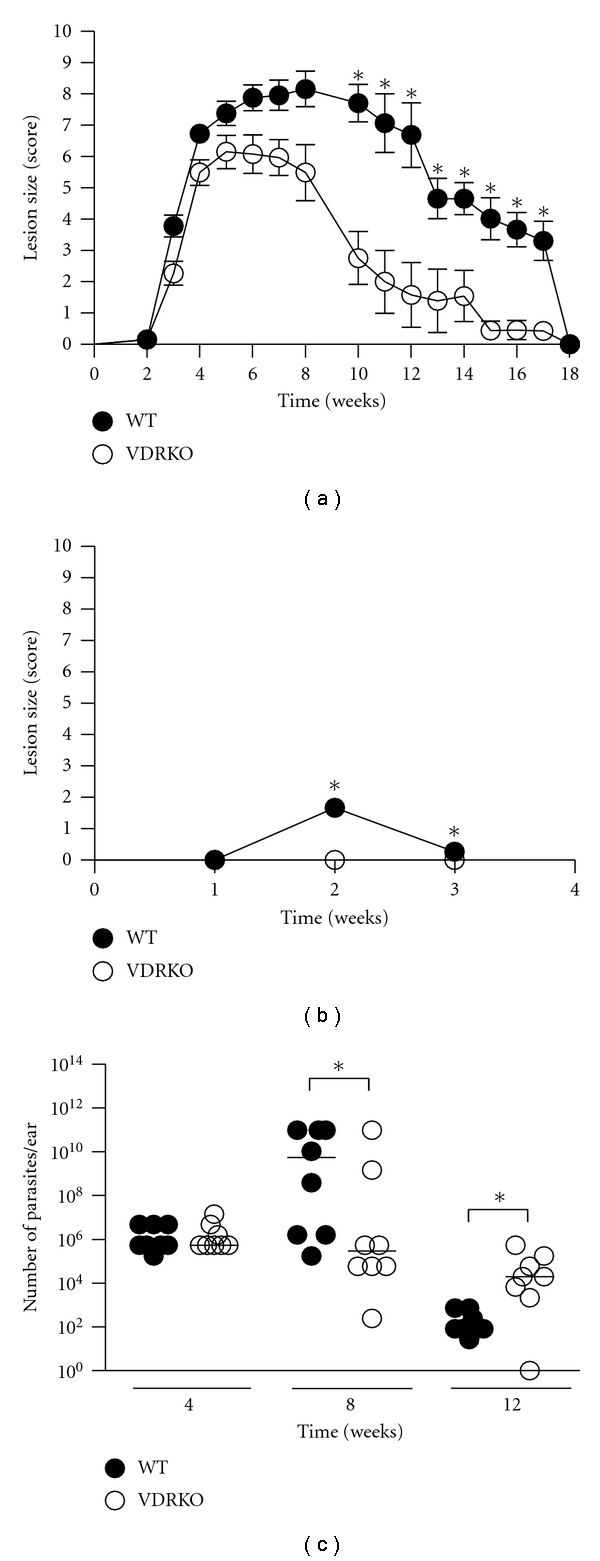
VDRKO mice develop smaller lesions when infected with *L. major*. C57BL/6 WT (closed circles) and VDRKO (open circles) mice were infected in the ear with 10^5^ *L. major* parasites. (a) Cumulative measurement of lesion size (score) was measured over the course of infection. One representative of 5 independent experiments is presented; *P ≤ 0.05. (b) Mice were reinfected upon complete resolution of the primary infection (wk 18) and lesions were measured until completely healed. Mean lesion score ± SE is presented; *n* = (11–21 ears). One representative of 2 independent experiments is presented. (c) Parasite burden of infected ears from VDRKO and WT mice was determined at 4, 8, and 12 weeks after infection by performing a limiting dilution assay. Each point indicates the parasite load in a single ear. One representative of 2 independent experiments is presented. *P ≤ 0.05.

 To investigate the role of VDR in immunity to *L. major *infection, C57BL/6 VDRKO and WT mice were infected in the ear dermis with 10^5^ *L. major* metacyclic promastigotes. Both groups of mice developed lesions that eventually healed; however, VDRKO mice developed lesions that were significantly smaller and healed 3 weeks faster than their WT counterparts (Figure 1(a)). Upon reinfection, VDRKO mice did not develop lesions at the site of secondary infection whereas the WT mice developed small lesions that resolved within 3 weeks (Figure 1(b)). These results indicate that VDRKO mice have an increased resistance to *L. major* infection compared to WT mice as demonstrated by decreased lesion development. Additionally, VDRKO mice have an intact and possibly heightened memory response to secondary infection.

Because VDRKO mice had decreased lesion development, we compared the parasite load at the site of infection between VDRKO and WT mice using a limiting dilution assay. At 4 weeks after infection, both groups of mice had similar levels of parasites at the site of infection and at week 8, the VDRKO mice had significantly fewer parasites compared to the WT mice (Figure 1(c)). However, by 12 weeks after infection, VDRKO mice harbored significantly elevated parasite loads, suggesting that wound healing and parasite killing may be occurring via different mechanisms [20].

### 3.2. 1-*α* Hydroxylase-Deficient Mice Exhibit Heightened Resistance to L. major Infection

**Figure 2:**
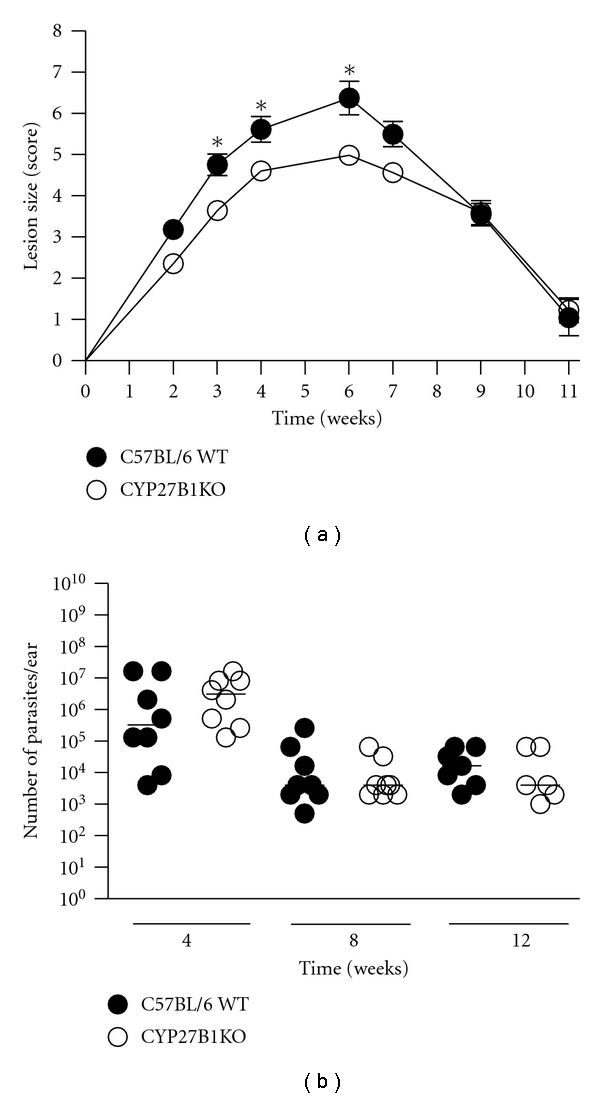
Lesion size is reduced in CYP27B1KO mice. C57BL/6 WT (closed circles) and CYP27B1KO (open circles) mice were infected with 10^5^ *L. major* parasites and the resulting lesions were measured at the indicated time points. Mean ± SE is presented; *n* = (8–17). (b) Infected ears were harvested at 4, 8, and 12 weeks after infection, and parasite load was determined by limiting dilution assay. Each point indicates parasite burden in one ear. One representative of two independent experiments is presented. *P ≤ 0.05.

CYP27B1KO mice, which lack the 1-*α* hydroxylase enzyme required to produce 1,25D3, displayed prototypical lesion development that is observed in resistant (C57BL/6) strains of mice; that is, lesions develop after a few weeks and eventually resolve. Similar to VDRKO mice, CYP27B1KO mice developed significantly smaller lesions compared to their WT counterparts throughout the first 6 weeks of infection (Figure 2(a)). CYP27B1KO mice resolved their infections at a similar rate as WT mice and both groups were completely healed by week 11 after infection. Parasite quantification of infected ears from these groups of mice demonstrated that that CYP27B1KO and WT mice possess similar parasite burdens throughout the infection (Figure 2(b)).

### 3.3. VDR Ablation Does Not Affect *L. major* Infection in BALB/c Mice

**Figure 3:**
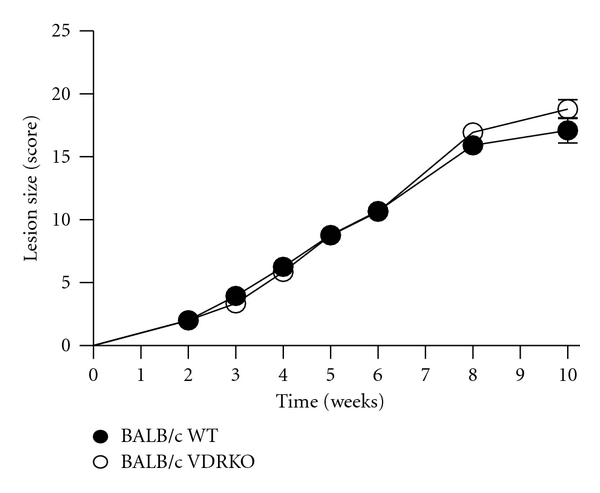
Deficiency of vitamin D signaling does not modulate resistance in a susceptible mouse strain. VDRKO mice on a susceptible BALB/c background (open circles) or WT BALB/c (closed circles) were infected with 10^5^ *L. major* parasites and the resulting lesions were measured at the indicated time points. Mean ± SE is presented; *n* = 14. One representative of two independent experiments is presented.

C57BL/6 mice have the propensity to completely heal after *Leishmania* infection, whereas BALB/c mice are unable to heal or resolve *L. major* induced lesions. Unlike C57BL/6 VDRKO mice, VDRKO mice on a susceptible BALB/c background did not develop smaller lesions compared to their WT counterparts (Figure 3).

### 3.4. VDRKO Mice Exhibit Decreased Inflammation at the Site of Infection

**Figure 4:**
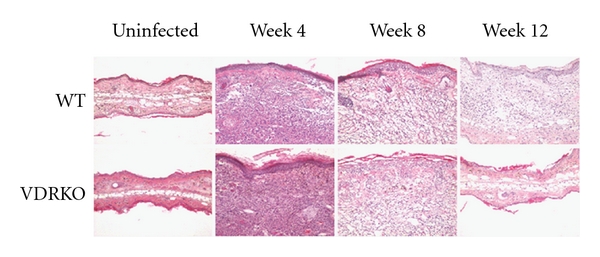
Inflammation is decreased in the infected ears of VDRKO mice. C57BL/6 WT and VDRKO were infected in the ear with 10^5^ *L. major* parasites. Histological cross sections were H&E stained at the indicated time points and inflammation of the dermis was assessed visually by microscopy. One representative of 2 independent experiments is presented.

To investigate the causes of reduced lesions and parasite burden in C57BL/6 VDRKO mice, we performed further analyses on lesions and immune responses to *L. major *infection. VDRKO mice exhibit alopecia, develop dermal cysts, and do not recycle epidermal layers properly [21, 22]. To assess if these skin differences were involved in the differential lesion development, we performed a histological study to explore lesion architecture. Uninfected C57BL/6 VDRKO and WT mice do not have any differences in their skin architecture and displayed no characteristics indicative of inflammation (Figure 4). At 4 weeks after infection, both VDRKO and WT mice exhibit similar levels of inflammation at the site of infection. Although numbers of infiltrating cells were similar in the two mouse strains through 8 weeks of infection (data not shown), VDRKO mice appeared to display decreased inflammation by 8 weeks after infection (Figure 4). Inflammation continued to decrease in both groups of mice, and by 12 weeks after infection lesions from VDRKO mice exhibited little signs of inflammation. Inflammation was still observed in the WT mice at 12 weeks after infection although greatly reduced. 

There were no significant differences between WT and VDRKO mice in terms of macrophage (CD11b+/F480+), neutrophil (Ly-6C/G+/F480-), T cell (CD4+ or CD8+), or dendritic cell (CD11c+) cell infiltration (data not shown).

**Figure 5:**
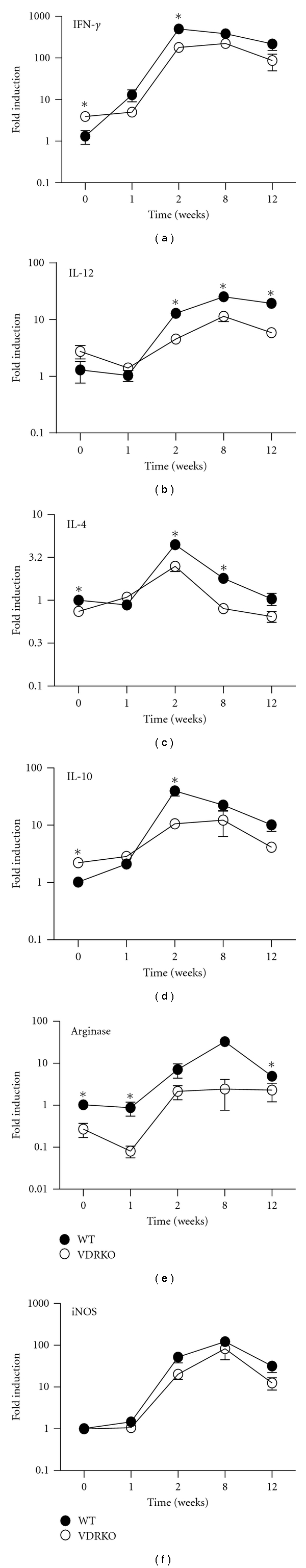
Quantitative RT-PCR analysis of cytokine production at the site of infection. C57BL/6 WT (closed circles) and VDRKO (open circles) mice were infected with 10^5^ *L. major* parasites and their ears were harvested at the indicated time points. Quantitative RT-PCR was performed to determine the fold increase over uninfected WT ears for each cytokine. One representative of two independent experiments is presented. *P ≤ 0.05. *n* = 4 mice/time point.

WT mice produced more IFN*γ* and IL-10 mRNA at the infection site 2 weeks after *L. major* infection (Figure 5). In addition, IL-4 and IL-12 mRNA was elevated in WT mice compared to VDRKO mice. Inducible nitric oxide synthase (iNOS) was upregulated during *L. major* infection, however no differences between WT and VDRKO were detected. As previously reported [17], WT mice express more arginase mRNA than VDRKO mice (Figure 5).

### 3.5. Systemic Immune Responses in VDR KO and WT Mice

Flow cytometry analysis of the draining lymph nodes indicated that T-helper cells (CD4+), cytotoxic T-cells (CD8+), and FoxP3+ Treg (CD4+/CD25+) cells are elevated in VDRKO mice at most time points relative to WT animals (Suppl. Figure  1 which is available online at doi:10.1155/2012/134645). The number of each of these cell types increased as the lesions progressed and then decreased during healing. No obvious differences in other cell types such as macrophages (CD11b+/F480+), dendritic cells (CD11c+), or neutrophils (LY-6C/G+/F480-) were observed in the draining lymph nodes (data not shown). 

VDRKO lymph node cells produced significantly lower amounts of inflammatory cytokines IFN*γ*, TNF-*α*, GM-CSF, and MIP-1*α* at the height of infection than the cells from WT mice (Suppl. Figure  2). Conversely, more MCP-1 was produced by lymph node cells from VDRKO mice compared to the cells from WT mice. Early after infection (2 weeks), cells from both mouse strains up regulated IL-4, IL-5, and IL-13, production that decreased by 12 wks post-infection (Suppl. Figure  2). Furthermore, VDRKO and WT lymph nodes generated equivalent amounts of IL-1*β*, IL-6, IL-10, Rantes, and KC at all time points. 

**Figure 6:**
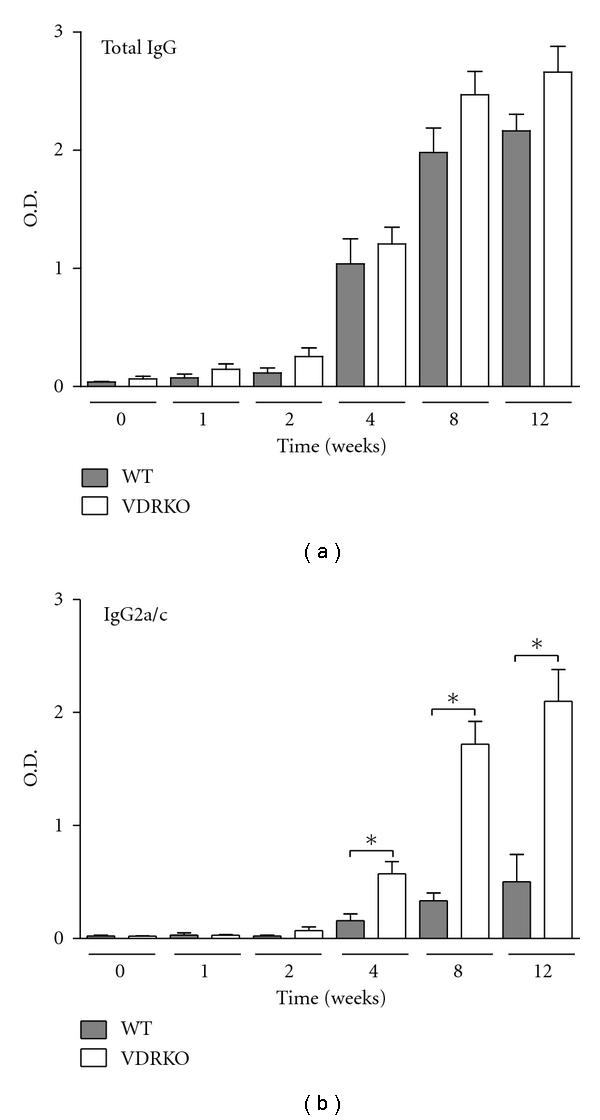
VDRKO mice exhibit elevated IgG2a/c production in response to *L. major* infection. C57BL/6 WT (closed bars) and VDRKO (open bars) mice were infected with 10^5^ *L. major* parasites and serum samples were collected at the indicated time points. Isotype specific ELISA was performed to determine levels of total IgG and IgG2a/c in the serum. Mean ± SE is presented; *n* = (8–10). *P ≤ 0.05. One representative of two independent experiments is presented.

Total IgG serum levels increased in both groups of mice as lesion development progressed and remained elevated even after the mice had healed (Figure 6). Significantly elevated levels of IgG2a/c were detected in VDRKO mice beginning at week 4 after infection and remained significantly elevated throughout infection (Figure 6). No differences in IgG1 titers were observed between the VDRKO and WT mice at any time point (data not shown).

### 3.6. NO and IL-12 Production Are Increased in DC from VDRKO C57BL/6 Mice

**Figure 7:**
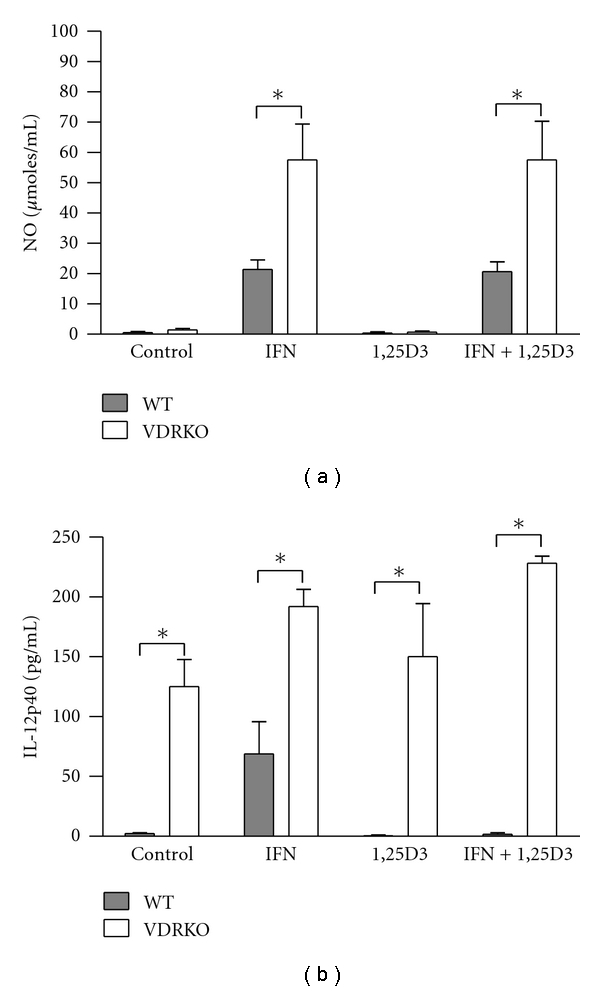
NO and IL-12p40 production by host cells. C57BL/6 WT (closed bars) and VDRKO (open bars) mice. DC were treated with IFN*γ* and/or 1,25D3 for 24 hr prior to being infected at a ratio of 5 : 1 with *L. major. *The cells were infected for 4 hr and washed to remove extracellular parasites. Supernatants were harvested 48 hr after infection and analyzed for production of NO (a) and IL-12p40 (b). Mean ± standard error is presented; (*n* = 4). *P ≤ 0.05.

NO production is a potent mechanism used by antigen presenting cells to eliminate *L. major* parasites. DC from both groups of mice were either left untreated or preincubated with combinations of IFN-*γ* and/or 1,25D3 prior to *L. major* infection and 48 hours after infection, cell culture supernatant was analyzed for levels of NO production. DC from VDRKO generate significantly more NO upon stimulation with IFN*γ* or IFN*γ* and 1,25D3 (Figure 7(a)). Preincubation of IFN*γ* or a combination of IFN*γ* and 1,25D3 resulted in significantly more NO production as compared to untreated cells in both cell types. We did not observe an inhibition of NO production by the DC upon treatment with 1,25D3 in either the VDRKO or WT derived cells. In addition, there is no effect of either the VDR or 1,25D3 on NO production in macrophages (Suppl. Figure  3(a)).

Regardless of the IFN*γ* and/or 1,25D3 treatment, IL-12p40 production by VDRKO DC is significantly increased compared to WT DC, suggesting that VDR may contribute to regulation of IL-12p40 production by DC (Figure 7(b)).

## 4. Discussion

*Leishmania* are obligate intracellular parasites that are eliminated by a strong Th-1 host response. Vitamin D treatment reduces inappropriate Th-1 responses thus decreasing or eliminating symptoms of autoimmune diseases [6–8, 23]. As vitamin D exerts these effects through the VDR, we hypothesized that ablation of the VDR would tilt the Th-1/Th-2 balance towards a Th-1 bias and lead to enhanced resistance to *L. major* infection. Using a mouse model of cutaneous leishmaniasis, in which the parasites were injected into the ear dermis, we observed that VDRKO mice developed significantly smaller lesions and have fewer parasites at the site of infection during the height of infection than their WT counterparts. These results are similar to those observed by previous studies using a foot pad model of cutaneous leishmaniasis [17]. As VDRKO mice healed earlier than WT mice, we anticipated that VDRKO mice would exhibit decreased parasite loads compared to WT mice, because VDRKO mice resolve their lesions more quickly than WT mice. However, at 12 weeks after infection, VDRKO mice had elevated levels of parasites at the infection site even though they have decreased lesion size. This disparity may indicate that different processes are contributing to wound healing and parasite killing. The ability of VDRKO mice to produce increased dermal depositions of collagen may contribute to the increased healing phenotype we observed in VDRKO mice, as research indicates that orderly collagen fiber deposition in the skin is one factor that may contribute to increased healing in *L. major* infected mice [20].

Successful clearance of a *L. major* infection depends on initiation of a robust Th-1 response that leads to parasite killing via production of NO. VDRKO mice produce significantly lower levels of inflammatory cytokines locally at the infection site and following restimulation in vitro suggesting that VDRKO mice generate a decreased Th-1 response to *L. major *infection. We expected to detect upregulated transcription of iNOS in VDRKO mice as this enzyme leads to production of the NO necessary for killing of *L. major*; however, iNOS is not upregulated at any point post *L. major* infection in VDRKO compared to WT mice. Rather, arginase transcript levels are higher in WT compared to VDRKO mice, supporting the suggestion by others that upregulation of arginase antagonizes the metabolism of NO by competing with iNOS for a common substrate, L-arginine [17]. The lack of such competition would allow VDRKO mice to generate more NO than WT mice, leading to increased parasite killing. This hypothesis is further supported by studies demonstrating that inhibition of arginase by N-hydroxy-L-arginine, a precursor of NO, results in increased macrophage killing of *L. major* parasites *in vitro*, while induction of arginase contributes to the growth of *L. major* by providing the parasites with the polyamines required for replication [24, 25]. 

Toll like receptor activation initiates upregulation of VDR and CYP27B1 leading to an antimicrobial peptide response against *Mycobacteria* [10, 26], which contributes to killing of *M. tuberculosis* in human macrophages [27]. This pathway is unlikely to play a role in resistance to *L. major *as antimicrobial peptides have little effect in killing of *Leishmania *[28, 29]. VDR expression, treatment with IFN*γ* and/or vitamin D does not affect the ability of macrophage and DC to phagocytose *L. major *(data not shown and [17]). Furthermore, 1,25D3 treatment alone had no effect on IFN*γ*-induced NO production in infected macrophage and DC in vitro. This contrasts with other published data suggesting that NO production by *L. major* infected macrophages is inhibited by 1,25D3 [17]. These authors suggest that enhanced production of arginase competes with iNOS for a common substrate, ultimately resulting in decreased production of NO in macrophage. Stimulation of VDRKO macrophage with IFN*γ* and LPS resulted in significantly less NO than similarly treated WT macrophage (data not shown) suggesting that VDR may play a role in modulation of NO production in macrophage. Conversely, IFN*γ*-induced NO production by infected VDRKO DC was significantly higher than WT DC implying that VDR ablation increases rather than decreases NO production in DC. Our data suggests that vitamin D and the VDR differentially regulate NO production in macrophage versus DC. 

In addition to overproducing NO, VDRKO DC overproduce IL-12p40 compared to WT. Other studies demonstrate that 1,25D3 inhibits IFN*γ* signaling in both T cells, macrophages and DC [17, 30–33]. Indirect inhibition of DC produced NO then could result via VDR inhibition of IFN*γ* activated STAT1 signaling. The ablation of the VDR results in elimination of this inhibition resulting in upregulation of NO and IL-12p40 production. As a similar increase of IL-12p40 and NO production was not observed in VDRKO macrophage this implies that macrophage differ from DC in their IFN*γ* induced production of IL-12p40.

VDRKO mice generate significantly more antigen specific IgG2a/c antibodies, that serve as an indicator of Th-1 biased immune responses [34, 35] than WT mice. The elevated IgG2a/c production in VDRKO mice is not surprising as treatment with 1,25D3 inhibits IgG2a production in mice [36], in cattle [37], and in pigs [38]. These data suggest that elimination of inhibitory effects of 1,25D3 skews antibody response towards production of IgG2a/c. The higher levels of IgG2a/c possibly explain the increased resistance to secondary infection with *L. major* observed in the VDRKO mice (Figure 1(b)). Indeed, elevated antigen specific and nonspecific induction of IgG2a/c has been observed in older VDRKO mice infected with *Listeria monocytogenes *[39]. However, the exact relationship between IgG2a/c production and vitamin D signaling remains to be elucidated.

We demonstrate that neither genetic removal of the VDR or depletion of vitamin D from the diet (data not shown) of mice on the BALB/c background does not alter susceptibility to *L. major* infection. Susceptibility in WT BALB/c mice is attributed to their inability to mount a Th-1 response against infection. Our results indicate that the effect of the VDR may depend on the nature of the host immune response. Additional mechanisms, such as wound healing and genetic background, have also been shown to be important in resistance to *L. major* infection [20, 40]. Our data clearly show that the absence of vitamin D signaling cannot overcome the susceptibility traits of BALB/c mice.

Contrary to the results observed in VDRKO or CYP27B1KO mice, dietary deficiency of vitamin D in resistant C57BL/6 mice did not reduce the severity of *L. major* lesions (data not shown). A similar disparity has been observed in studies investigating the role of VDR deletion versus dietary vitamin D deficiency in the development of diabetes [23, 41] and MS [8, 42]. Our data may indicate that some of the effects of VDR on the course of *L. major* infection are ligand independent, as has been shown in the case of alopecia [43], or that sufficient 1,25D3 persists in mice maintained on the deficient diet to activate the receptor. 

In summary, we have demonstrated that VDRKO mice on the C57BL/6 background develop smaller lesions than WT mice upon infection with *L. major*, but this phenotype is not observed on the BALB/c genetic background. Enzymatic depletion of 1,25D3 also enhances resistance to *L. major* infection in C57BL/6 mice. The data further suggest that increased IL-12p40 and NO production by VDRKO dendritic cells may contribute to increased resistance to *L. major*. These studies indicate that ablation of VDR or elimination of its ligand, 1,25D3, is able to increase resistance to *L. major* infection, but only in a host that is predisposed for Th-1 immune responses. Our data confirm an important role for vitamin D for regulating immune responses that depend on Th-1 cells.

## Supplementary Material

 VDRKO mice have increased numbers of T-cells in the draining lymph nodes; restimulation of lymph nodes; nitric oxide and IL-12p40 by macrophages from WT and VDRKO mice.
