# Supplementary material for: The Role of Vitamin D and Vitamin D Receptor in Immunity to Leishmania major Infection
Source: J Parasitol Res. 2011 Oct 11;2012:134645. doi: 10.1155/2012/134645 (PMC3191735; doi:10.1155/2012/134645)
Supplement: Supplementary file 1 — VDRKO mice have increased numbers of T-cells in the draining lymph nodes; restimulation of lymph nodes; nitric oxide and IL-12p40 by macrophages from WT and VDRKO mice. [file 134645.f1.pdf]

SUPPLEMENTAL FIGURE LEGENDS

Supplemental Figure 1. VDRKO mice have increased numbers of T-cells in the draining lymph nodes. C57BL/6 WT (closed bars) and VDRKO (open bars) mice were infected with 10^5 *L. major* parasites and their submandibular lymph nodes were harvested at the indicated time points. Lymph nodes were homogenized, pooled (n=4), and analyzed by flow cytometry. Numbers of (A) T-helper cells (TCR+/CD4+), (B) Cytotoxic T-cells (TCR+/CD8+), and (C) T-regulatory cells (CD4+/CD25+/FoxP3+) were calculated. One representative of two independent experiments is presented.

Supplemental Figure 2. Restimulation of lymph nodes. C57BL/6 VDRKO and WT mice were infected in the ears with 10^5 *L. major* parasites. Lymph nodes were harvested and homogenized at the indicated time points. Cell homogenates were plated in 96 well plates and restimulated with soluble *Leishmania* antigen. The supernatants were analyzed by multi-analyte immunoassay for cytokines representative of Th-1 and Th-2 responses. One representative of two independent experiments is presented. $^*p \leq 0.05$. n = 4 mice/time point.

Supplemental Figure 3. Nitric oxide and IL-12p40 by macrophages from WT and VDRKO mice. Macrophages were treated with IFNγ and/or 1,25D3 for 24 hr prior to being infected at a ratio of 5:1 with *L. major.* The cells were infected for 4 hr, washed to remove extracellular parasites. Supernatants were harvested 48 hr post-infection and analyzed for production of NO (A) and IL-12p40 (B). Mean ± standard error is presented; (n=4). $^*p \leq 0.05$.

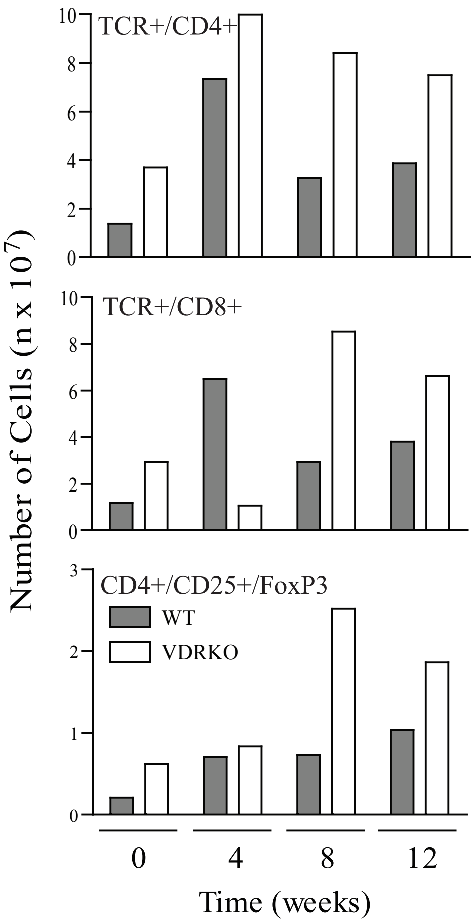

Supplemental Figure 1

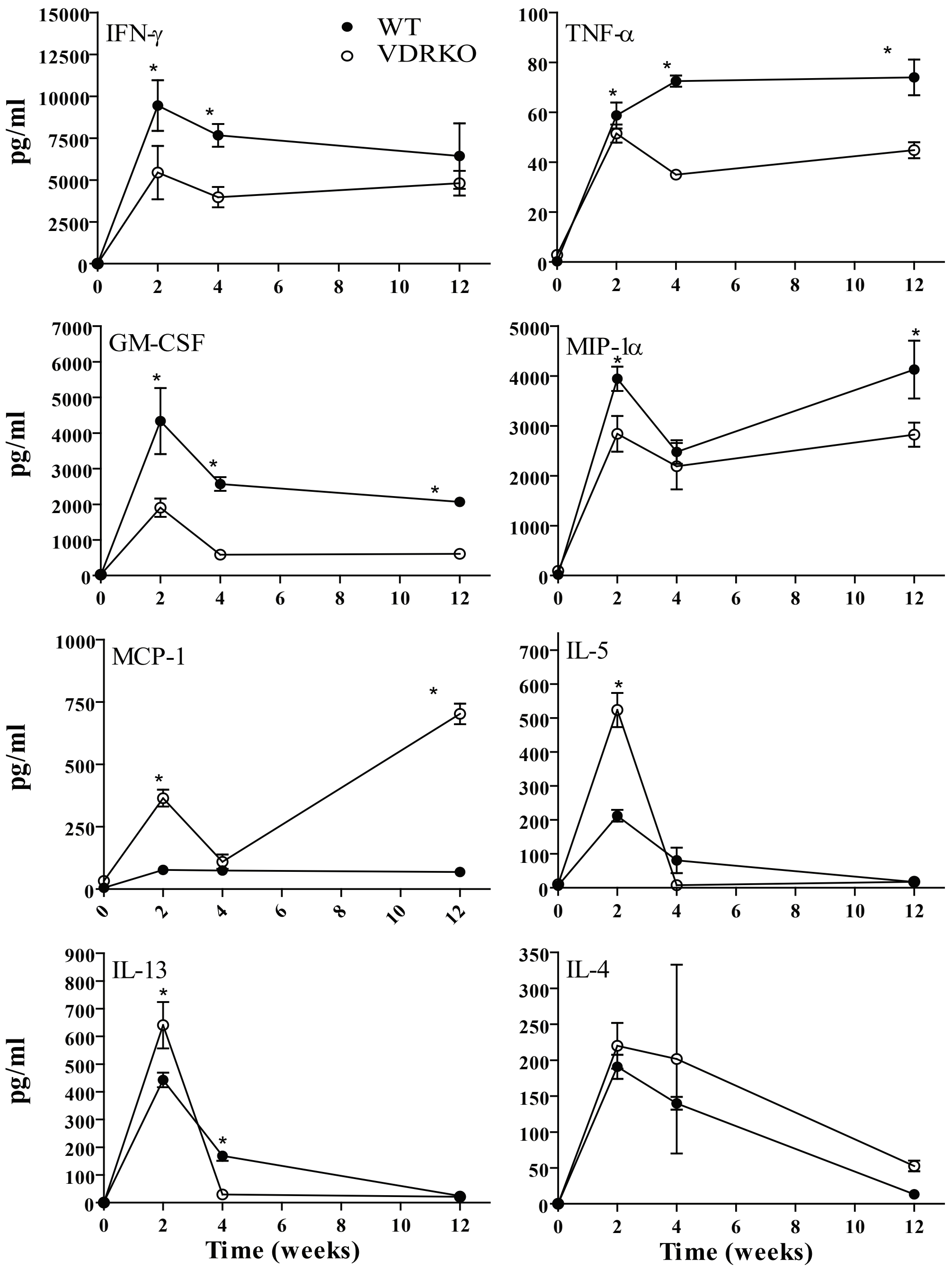

Supplemental Figure 2

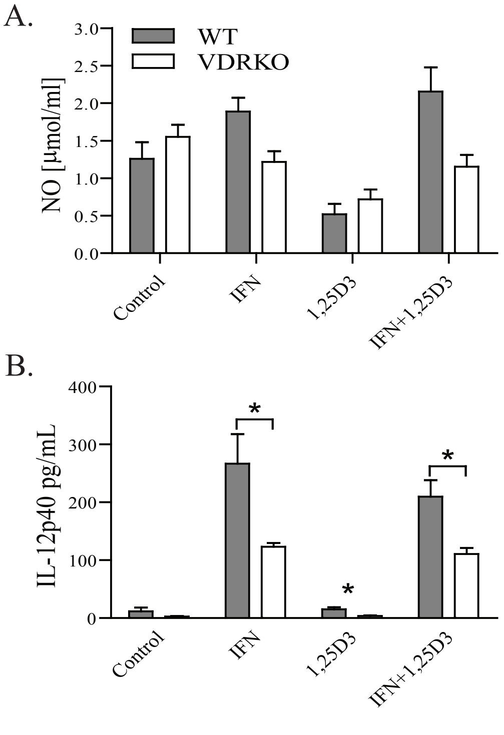

Supplemental Figure 3
